# Enzymes of the one-carbon folate metabolism as anticancer targets predicted by survival rate analysis

**DOI:** 10.1038/s41598-017-18456-x

**Published:** 2018-01-10

**Authors:** Jun Koseki, Masamitsu Konno, Ayumu Asai, Hugh Colvin, Koichi Kawamoto, Naohiro Nishida, Daisuke Sakai, Toshihiro Kudo, Taroh Satoh, Yuichiro Doki, Masaki Mori, Hideshi Ishii

**Affiliations:** 1 Department of Medical Data Science, Osaka University, Osaka, 565-0871 Japan; 2 Department of Frontier Science for Cancer and Chemotherapy, Osaka University, Osaka, 565-0871 Japan; 3 Department of Gastroenterological Surgery, Graduate School of Medicine, Osaka University, Osaka, 565-0871 Japan

## Abstract

The significance of mitochondrial metabolism in cancer cells has recently been gaining attention. Among other findings, One-carbon folate metabolism has been reported to be closely associated with cellular characteristics in cancer. To study molecular targets for efficient cancer therapy, we investigated the association between the expressions of genes that code enzymes involved in one-carbon metabolism and survival rate of patients with adenocarcinomas of the colorectum and lung. Patients with high expression of genes that control the metabolic cycle of tetrahydrofolate (THF) in mitochondria, *SHMT2*, *MTHFD2*, and *ALDH1L2*, have a shorter overall survival rate compared with patients with low expression of these genes. Our results revealed that these genes could be novel and more promising anticancer targets than dihydrofolate reductase (DHFR), the current target of drug therapy linked with folate metabolism, suggesting the rationale of drug discovery in cancer medicine.

## Introduction

Folate is very important in bioorganic systems, although it cannot be synthesized in humans^1^. It is acquired from food and converted to tetrahydrofolate [tetrahydrofolic acid (THF)], which is the reactant in the metabolic pathway of the folate cycle. The folate cycle has a crucial role in supplying one-carbon (C1) groups (e.g., methyl, methylene, and methenyl groups) that are transferred to biomolecules, such as amino acids and nucleotides, which explains why the metabolism of the folate cycle is also known as C1 metabolism^2–9^. C1 metabolism plays a role in DNA synthesis and repair via production of nucleic acid components^5,6^. In the first step of C1 metabolism, some of the folate is converted to an intermediate metabolite, dihydrofolate (DHF), by a reduction reaction catalyzed by an enzyme, dihydrofolate reductase (DHFR), and the rest is directly converted to THF. THF is extensively used as a source of intermediates in other reaction pathways involved in C1 metabolism^7–10^. DHF is also converted to THF with time.

Recently, C1 metabolism has received much attention as a target of cancer therapy, and many studies have reported it to be a promising target for cancer treatment^11–13^. Many drugs have been developed to target folate metabolism by inhibiting DHFR. However, folate metabolism is absolutely imperative not only for cancer cells but also for normal cells. Folate deficiency in humans results in the reduction of DNA synthesis and methylation^11,12^. Therefore, inhibition of DHFR is known to cause toxicity in normal cells as well as cancer cells.

**Figure 1:**
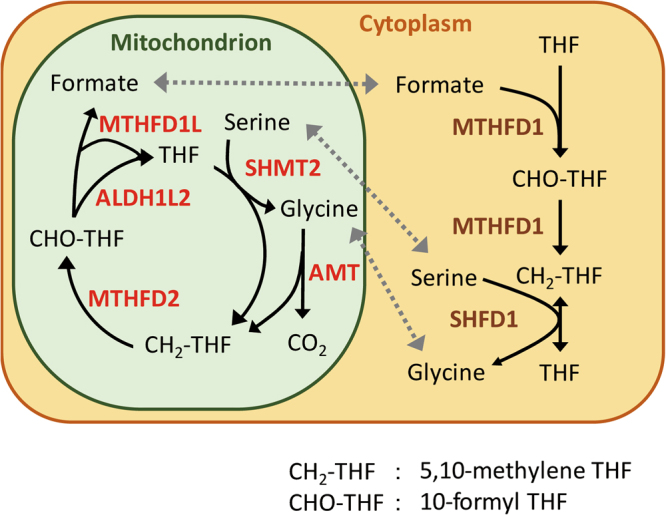
Schematic diagram of the metabolic cycle of THF and the C1 metabolic pathway.

In contrast, mitochondrial metabolism has received much attention as a potential target for cancer therapy^7,13^. We focused on the reactions among THF, 5,10-methylene THF (CH_2_-THF), and 10-formyl THF (CHO-THF) in C1 metabolism, which occur in the cytoplasm and mitochondria (Fig. 1). If folate metabolism in the mitochondria is responsible for poor prognosis in cancer patients, then the selective targeting of this pathway could be effective with few side effects because of the parallel pathway in the cytoplasm.

Herein we have performed computational analysis to investigate the association between transcriptome profiles of genes involved in C1 metabolism in patients with colorectal cancer (CRC) and lung adenocarcinoma (LA) and the overall survival (OS) rate of patients. We have previously reported the usefulness of transcriptome analysis in examining cellular metabolism and its close correlation with information gained from metabolome analysis^14^.

**Figure 2:**
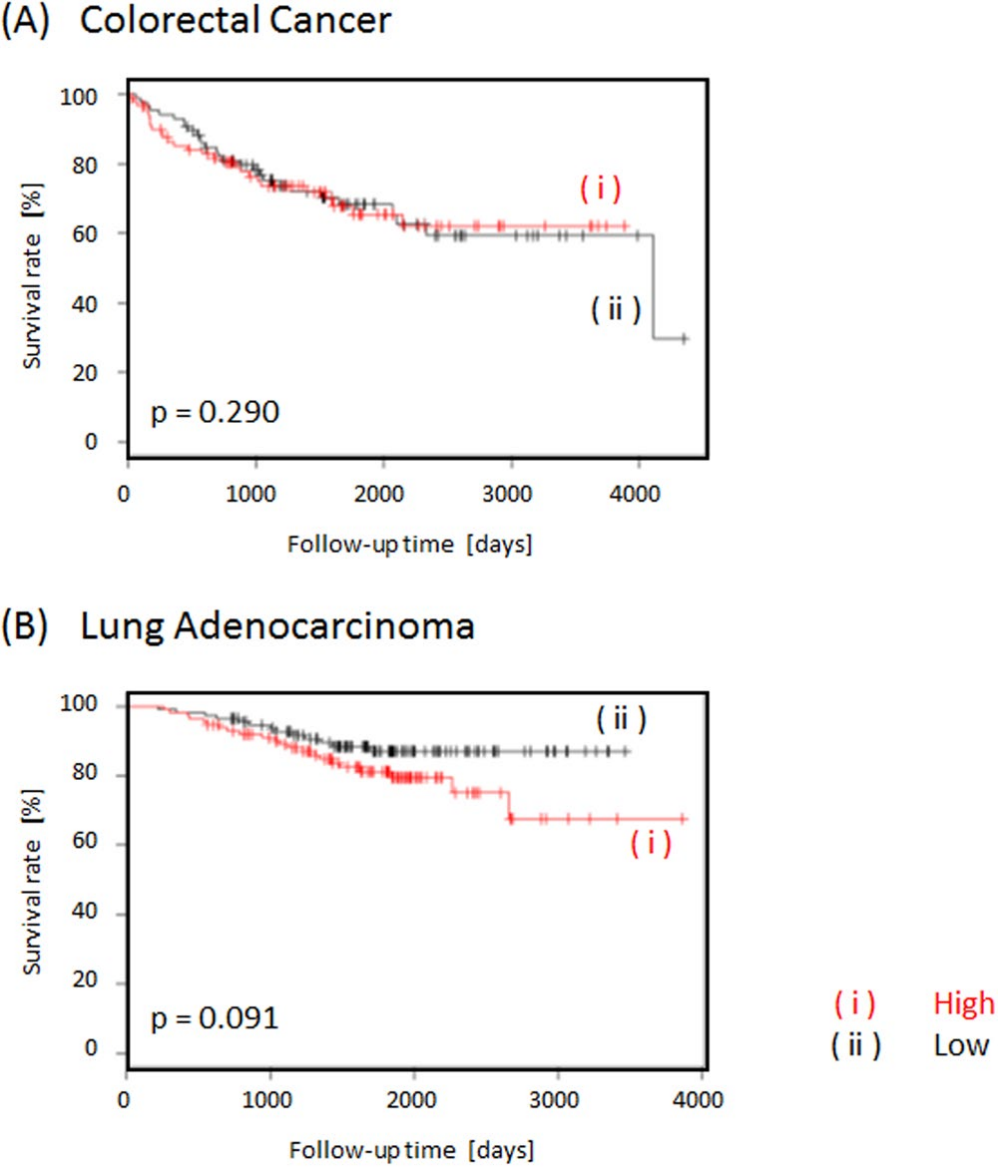
Kaplan–Meier curves of overall survival for (**A**) colorectal cancer and (**B**) lung adenocarcinoma according to the expression of DHFR, which is the conventional anticancer target of folate metabolism.

It is noteworthy that there was no strong association between the gene expression of *DHFR* and the OS of patients (Fig. 2). In contrast, we could identify other mitochondrial genes involved in C1 metabolism that are strongly associated with the prognosis in cancer patients, which we believe have the potential to be more efficient anticancer targets than *DHFR*, which is the anticancer target of folate metabolism.

## Results

**Figure 3:**
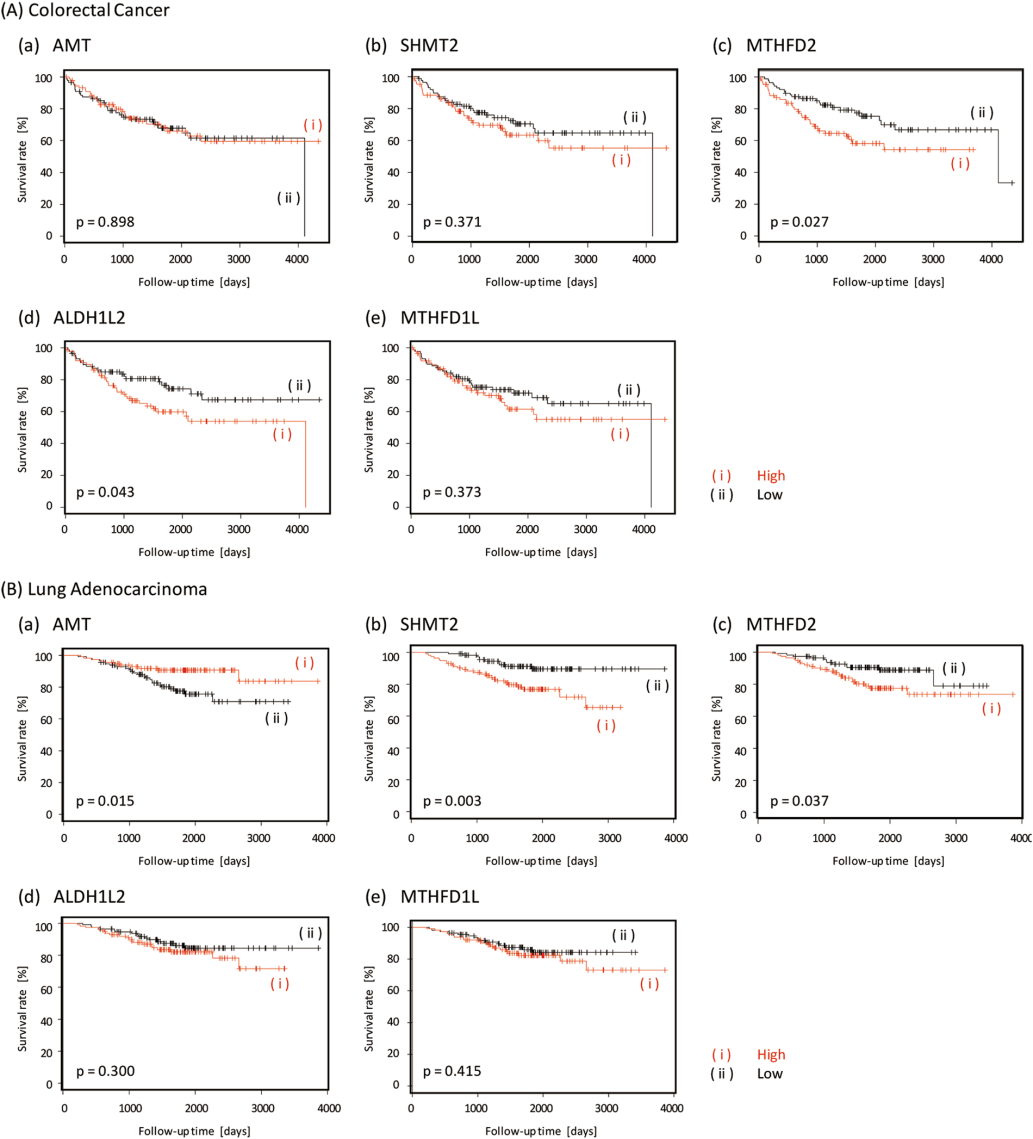
Kaplan–Meier curves of overall survival for (**A**) colorectal cancer and (**B**) lung adenocarcinoma according to the expression of genes involved in mitochondrial-specific folate metabolism (a) *ATM*, (b) *SHMT2*, (c) *MTHFD2*, (d) *ALDH1L2*, and (e) *MTHFD1L*.

Unexpectedly, the expression of *DHFR*, the conventional target for folate metabolism in cancer, did not dictate the OS in patients with CRC or LA. We thus searched for better anticancer targets of C1 metabolism and then performed correlation analysis between the expressions of genes involved in C1 metabolism and prognosis in patients with CRC and LA. In this study, we focused on five genes expressed in the mitochondria (*ATM*, *SHMT2*, *MTHFD2*, *ALDH1L2*, and *MTHFD1L*). The Kaplan–Meier curves for these genes in patients with CRC and LA are shown in Fig. 3. Although the expression of *ATM* did not affect the prognosis in patients with CRC, high expression of the gene was associated with a better prognosis in patients with LA (p = 0.015). In contrast, the high expression of *SHMT2* was associated with a significantly worse prognosis in patients with LA (p = 0.003), although the prognosis was not different in patients with CRC. The high expression of *MTHFD2* was associated with poor survival rates in patients with CRC (p = 0.027) and LA (p = 0.037). Similar to *SHMT2*, the high expression of *ALDH1L2* was associated with a significantly worse prognosis in patients with LA (p = 0.043), although the prognosis was not different in patients with CRC. The expression of *MTHFD1L* was not associated with patient prognosis. With the exception of *AMT*, we found that the high expression of four genes is associated globally with worse prognoses in patients with CRC and LA.

**Figure 4:**
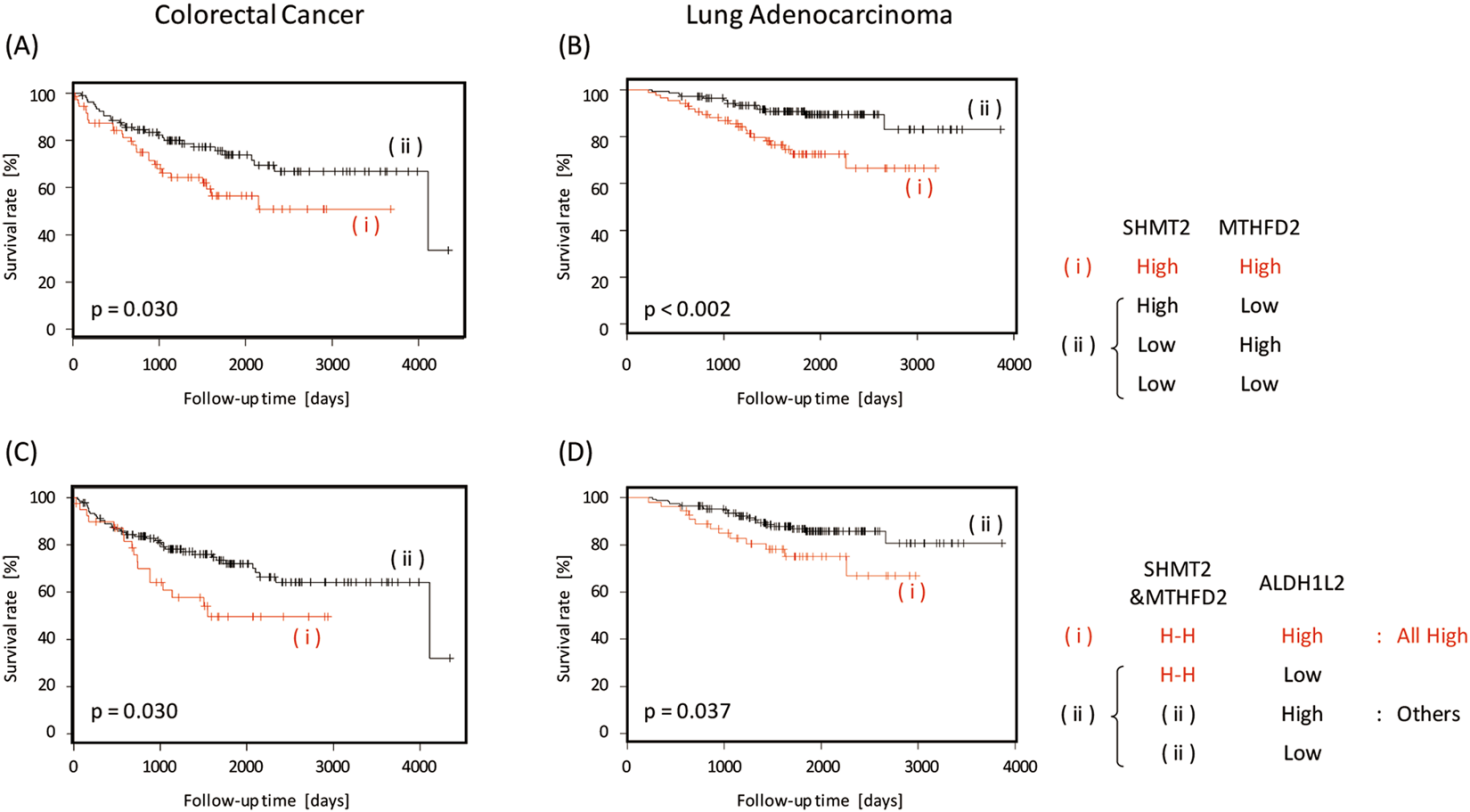
Correlation analysis of SHMT2, MTHFD2, and ALDH1L2, which are the enzymes involved in the THF cycle in the mitochondria. The Kaplan–Meier curves of the combination of the expression of SHMT2 and MTHFD2 for (**A**) colorectal cancer and (**B**) lung adenocarcinoma and of the combination of the expression of SHMT2, MTHFD2, and ALDH1L2 for (**C**) colorectal cancer and (**D**) lung adenocarcinoma.

From these results, we focused on the THF cycle in the mitochondria. We performed combination analyses to investigate the correlation among *SHMT2*, *MTHFD2*, and *ALDH1L2*, which all are involved in the THF cycle described above. Figure 4A (CRC) and 4B (LA) show the Kaplan–Meier curves for the combination of *SHMT2* with *MTHFD2*. Patients with high expressions of *SHMT2* and *MTHFD2* had worse prognoses compared to those with the other expression patterns of the enzymes in CRC (p = 0.030) and LA (p < 0.002). As shown in Supplementary Table 1, this behavior has a poor association with the age and sex of patients with CRC and LA. Additionally, according to Cox proportional hazards analysis and the likelihood ratio test, the p values were 0.032 and 0.034 for CRC, respectively, and 0.003 and 0.002 for LA, respectively. These results suggested that the prognosis could be improved in both cancers by decreasing the expression of either of the enzymes, SHMT2 or MTHFD2. In addition, patients with CRC and LA having high expressions of *SHMT2*, *MTHFD2*, and *ALDH1L2* had worse prognoses (Fig. 4C) (CRC), 4D (LA). Survival rate analysis also revealed that patients with high expression of all three enzymes had worse prognoses than the other patients with CRC (p = 0.030) and LA (p = 0.037). In this case, we performed the same statistical analyses. As shown in Supplementary Table 2, although there was bias in the distribution of sex between both groups, the poor association with patient age also was obtained for CRC and LA. According to Cox proportional hazards analysis and the likelihood ratio test, the p values were 0.027 and 0.033 for CRC, respectively, and 0.025 and 0.031 for LA, respectively.

**Figure 5:**
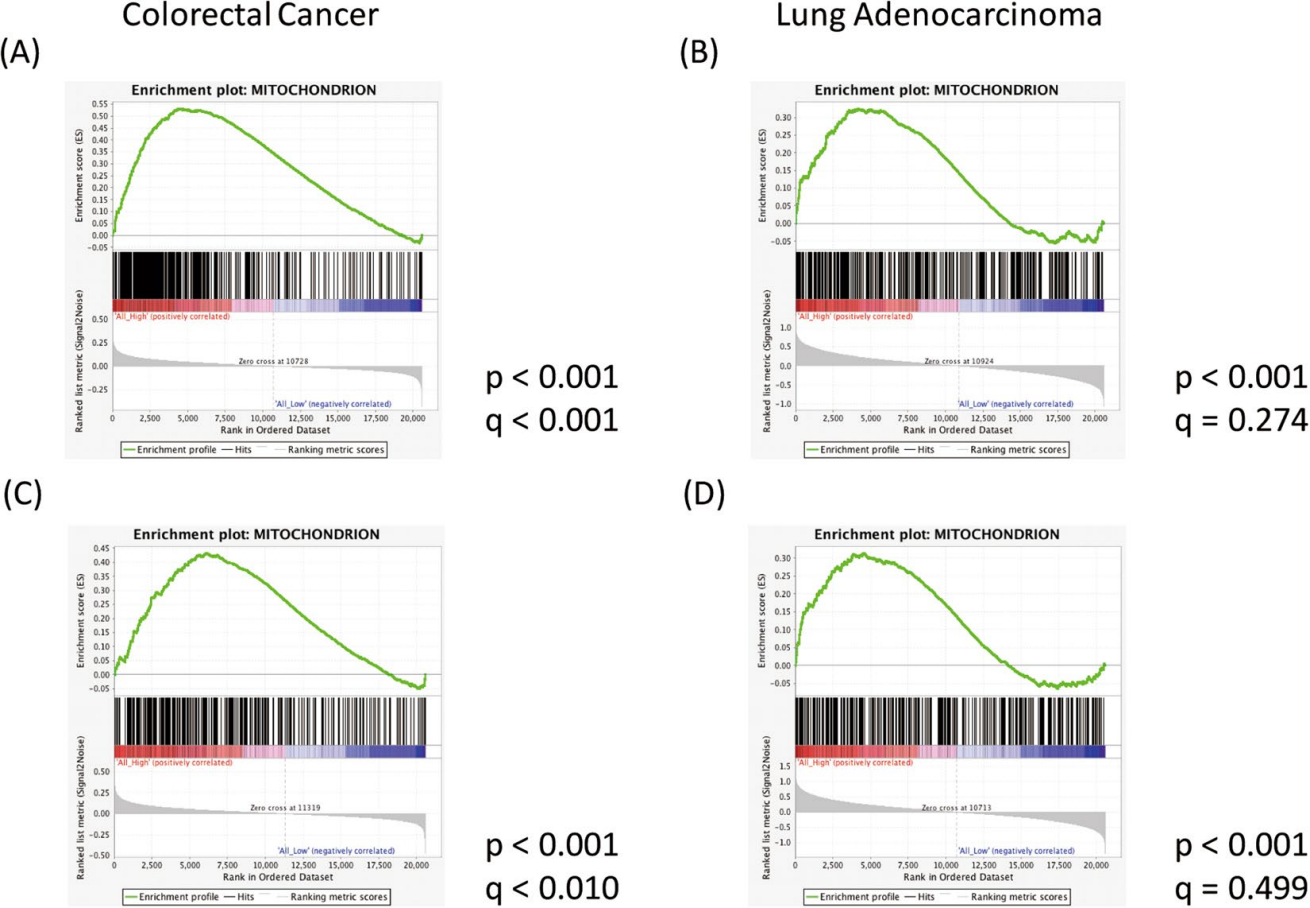
(**A**,**B**) GSEA analyses between the high-expression group and low-expression group in *SHMT2* and *MTHFD2* for colorectal cancer and lung adenocarcinoma, respectively. (**C**,**D**) Similarly, GSEA analyses were conducted between all high- and low-expression groups in *SHMT2*, *MTHFD2*, and *ALDH1L2*.

In addition, gene set enrichment analysis (GSEA) for genes involved in mitochondrial metabolism was performed to investigate whether the association between the THF cycle and cancer prognosis could be explained by the activity of the mitochondrial C1 metabolism. GSEA analyses revealed that, in CRC and LA, patients with high expression of SHMT2 and MTHFD2 had a high expression of genes associated with mitochondrial metabolism (Fig. 5A,B). Similarly, Fig. 5C and D show the GSEA results between all high- and low-expression groups for *SHMT2*, *MTHFD2*, and *ALDH1L2*. These figures also show the same tendency. These GSEA analyses revealed an association between the activities of the mitochondrial metabolism and the THF cycle.

We then investigated the association between the expression of genes involved in folic acid metabolism in the cytoplasm and prognosis. Intriguingly, the expression of *SHMT1*, *MTHFD1*, and *ALDH1L1* had no influence on the survival rate of patients with CRC and LA (Supplementary Figure 1), suggesting it is not the folate metabolism per se but the THF cycle in the mitochondria that confers poor prognosis in cancers.

**Figure 6:**
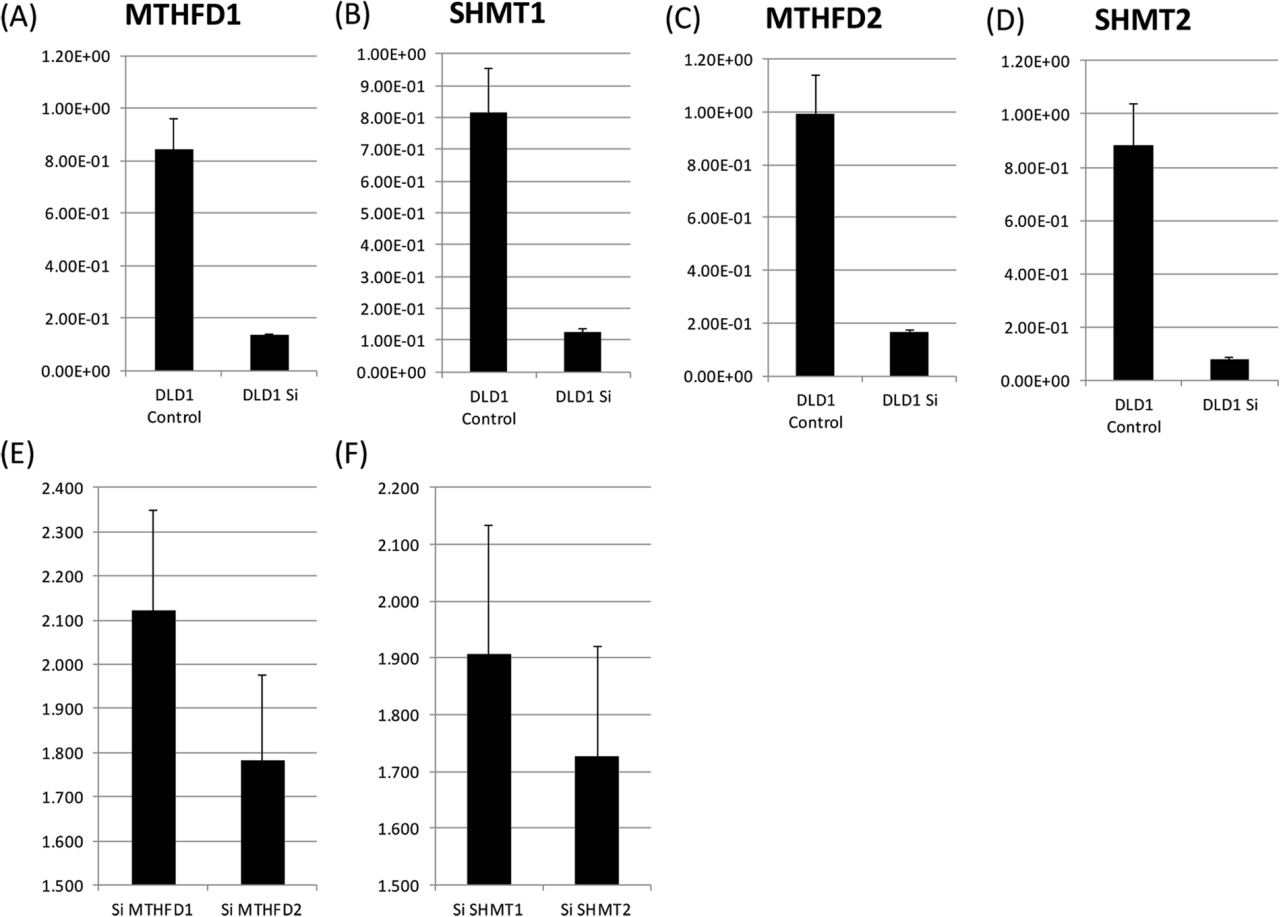
(**A**–**D**) Gene expression analysis for *SHMT1*, *MTHFD1*, *SHMT2*, and *MTHFD2*, under siRNA transfected condition. MTT assay under siRNA transfected conditions. (**E**) siMTHFD1 and siMTHFD2, and (**F**) SHMT1 and siSHMT 2.

Additionally, we have performed some siRNA knockdown experiments for cytoplasm enzyme (MTHFD1 and SHMT1) and C1 metabolic enzymes (MTHFD2 and SHMT2) at the *in vitro* level. As shown in Fig. 6A–D, the expression level of these mRNAs were downregulated by siRNA. Then, siRNA knockdown of SHMT2 and MTHFD2 resulted in more apparent inhibitory effect than SHMT1 and MTHFD1 (Fig. 6E,F). Moreover, we checked the expression of MTHFD2, SHMT2, and DHFR using the RefExA database. Although MTHFD2 and SHMT2 were highly expressed in colon cancer cells, DHFR had a very low or undetectable expression in colon cancer cells (Supplementary Figure 2). These data further suggest mitochondrial folate metabolism to be a promising target for cancer treatment.

## Discussion

Our study unexpectedly revealed that the expression of *DHFR*, the current anticancer target of folic acid metabolism, had no influence on the prognosis in patients with CRC or LA. Instead, we observed that the expression of genes controlling THF metabolism was associated with cancer prognosis, suggesting these genes to be more effective targets for cancer therapy than *DHFR*. Furthermore, we identified that the expression of genes was involved with only mitochondrial THF metabolism and not cytoplasmic THF metabolism, which was associated with a worse prognosis. Knockdown of the aforementioned enzymes in cancer cells confirmed that the mitochondrial enzymes involved in THF metabolism were crucial for cancer proliferation, but the cytoplasmic enzymes involved in THF metabolism were dispensable for cellular proliferation.

Specifically, we identified SHMT2 and MTHFD2 to be the most promising anticancer targets of folate metabolism from the correlational analyses between gene expression and survival. This may be because these enzymes are a crucial part of the THF cycle in mitochondria, and interference with their activities is predicted to create a bottleneck in the reaction cycle (Supplementary Figure 3).

Specific targeting of the mitochondrial folate metabolism would spare the physiological folate metabolism for occurring in the cytoplasm and thus may be associated with minimal side effects.

We also demonstrated the usefulness of correlational analysis between gene expression patterns and survival in cancers to identify novel targets of cancer metabolism. The novel targets identified by such analyses were validated by *in vitro* studies, which confirmed the usefulness of the correlational analysis in identifying potential targets.

We revealed novel anticancer targets of folate metabolism, which we predict will be associated with minimal toxicity given sparing of the physiological folate metabolism in the cytoplasm. Specifically, we predicted that SHMT2 and MTFD2 have the potential to be more effective anticancer targets than DHFR, which is the current anticancer target of folate metabolism.

## Materials and Methods

### Microarray Data of Gene Expression in Patients with CRC and LA

The GSE17536 database^15^ from Gene Expression Omnibus at the National Center for Biotechnology Information was used to analyze the effects of gene expression on the OS of patients (n = 177) with CRC, and the GSE31210 database^16^ was used for patients with LA (n = 226). These databases were generated using the Affymetrix Human Genome U133 Plus 2.0 Array. In these databases, the level of C1 expression is obtained with multiple probes for some genes. We selected the gene expression data for statistical analysis for probes that showed the widest variance of the expression for a particular gene and divided the expression data into low- and high-expression groups at the median. The GSE17536 and GSE31210 databases have data for patients of a broad generation aged around 60 years (range, 26–92 years and 30–76 years, respectively). The GSE17536 database reports gene expression for Stages I, II, III, and IV cancer. In contrast, the GSE31210 database has expression data for patients with only Stages I and II disease.

### Analysis of the Association between Gene Expression and Survival Rate

We focused on DHFR, the current anticancer target of folate metabolism, and five mitochondrial-specific genes involved in folate metabolism: *ATM*, *SHMT2*, *MTHFD2*, *ALDH1L2*, and *MTHFD1L*. For all of these genes, Kaplan–Meier curves for patients with CRC and LA was generated. Subsequently, combination analyses were performed to investigate the correlations among *SHMT2*, *MTHFD2*, and *ALDH1L2*, which are part of the THF cycle in mitochondria. In these analyses, we compared patients in the high-expression groups to those in the other groups. All Kaplan–Meier curves were generated using R package version 3.0.2^17^ with the survival package^18^.

### Cell Culture and MTT Assay

CRC cell line DLD1 was cultured in Dulbecco’s modified Eagle’s medium containing 10% fetal bovine serum. *SHMT1*, *MTHFD1*, *SHMT2*, and *MTHFD2* knockdown was performed using siRNA oligonucleotides synthesized from Sigma–Aldrich Corp. (St. Louis, MO, USA). After 36 h of culture from seeding, siRNAs were transfected into DLD1 cells at 20 nM final concentration with lipofectamine RNAiMax (Life Technologies, Carlsbad, CA, USA) with a forward transfection method according to the manufacturer’s protocol. MTT assay was performed using MTT (Nakarai Tesque) according to the manufacturer’s protocol.

### Gene Expression Analysis

Gene expression level was analyzed using the RefExA database (http://sbmdb.genome.rcast.u-tokyo.ac.jp/refexa/main_search.jsp).

## Electronic supplementary material

Supplementary Information

## References

[1] Stokstad ELR, Koch J (1967). Folic acid metabolism. Physiol. Rev..

[2] Coon MJ, Robinson WG (1958). Amino acid metabolism. Annu. Rev. Biochem..

[3] Jaenicke L (1964). Vitamin and coenzyme function: Vitamin B_12_ and Folic Acid. Annu. Rev. Biochem..

[4] Brown, G. M. Biogenesis and Metabolism of Folic Acid. (ed. Greenberg, D. M.) Chapter 24, 383–410, Metabolic Pathways, Vol. **IV** (Academic Press, New York. 1970).

[5] Duthie SJ (2004). Folate, DNA stability and colo-rectal neoplasia. Poc. Nutr. Soc..

[6] Jabrin S, Ravanel S, Gambonnet B, Douce R, Rébeillé F (2003). One-carbon metabolism in plants. Regulation of tetrahydrofolate synthesis during germination and seedling development. Plant Physiol..

[7] Christensen KE, MacKenzie RE (2006). Mitochondrial one-carbon metabolism is adapted to the specific needs of yeast, plants and mammals. BioEssays.

[8] Stover PJ, Field MS (2011). Trafficking of intracellular folates. Adv. Nutr..

[9] Momb J (2013). Deletion of Mthfd1l causes embryonic lethality and neural tube and craniofacial defects in mice. Proc. Natl. Acad. Soc. USA.

[10] Hsiao T-H (2014). Ethanol-induced upregulation of 10-formyltetrahydrofolate dehydrogenase helps relieve ethanol-induced oxidative stress. Mol. Cell. Biol..

[11] Dominguez-Salas P, Cox SE, Prentice AM, Hennig BJ, Moore SE (2012). Maternal nutritional status, C1 metabolism and offspring DNA methylation: a review of current evidence in human subjects. Proc. Nutr. Soc..

[12] Williams EA (2012). Folate, colorectal cancer and the involvement of DNA methylation. Proc. Nutr. Soc..

[13] Nilsson R (2014). Metabolic enzyme expression highlights a key role for MTHFD2 and the mitochondrial folate pathway in cancer. Nat. Commun..

[14] Koseki J (2016). A trans-omics mathematical analysis reveals novel functions of the ornithine metabolic pathway in cancer stem cells. Sci. Rep..

[15] Smith JJ (2010). Experimentally derived metastasis gene expression profile predicts recurrence and death in patients with colon cancer. Gastroenterology.

[16] Okayama H (2012). Identification of genes upregulated in ALK-positive and EGFR/KRAS/ALK-negative lung adenocarcinomas. Cancer Res..

[17] Team R. C. A language and environment for statistical computing. (R Foundation for Statistical Computing, Vienna, Austria 2013).

[18] Therneau, T. M. & Grambsch, P. M. Modeling Survival Data. Extending the Cox Model. (N.Y. Springer, 2000).

