# Supplementary material for: Enzymes of the one-carbon folate metabolism as anticancer targets predicted by survival rate analysis
Source: Sci Rep. 2018 Jan 10;8:303. doi: 10.1038/s41598-017-18456-x (PMC5762868; doi:10.1038/s41598-017-18456-x)
Supplement: Supplementary file 1 — Supplementary Information [file 41598_2017_18456_MOESM1_ESM.doc]

Enzymes of the one-carbon folate metabolism as anticancer targets predicted by survival rate analysis

Jun Koseki^1,†^, Masamitsu Konno^2,†^, Ayumu Asai^1,2^, Hugh Colvin^3^, Koichi Kawamoto^2,3^, Naohiro Nishida^3^, Daisuke Sakai^2^, Toshihiro Kudo^2^, Taroh Satoh^2^, Yuichiro Doki^3^, Masaki Mori^3^,*, Hideshi Ishii^1,2^,*

^1^ Department of Cancer Profiling Discovery, Osaka University, Osaka, 565-0871 Japan

^2^ Department of Frontier Science for Cancer and Chemotherapy, Osaka University, Osaka, 565-0871 Japan

^3^ Department of Gastroenterological Surgery Graduate School of Medicine, Osaka University, Osaka, 565-0871 Japan

^†^These authors contributed equally to this work.

*To whom correspondence should be addressed

**Supplementary Figure**


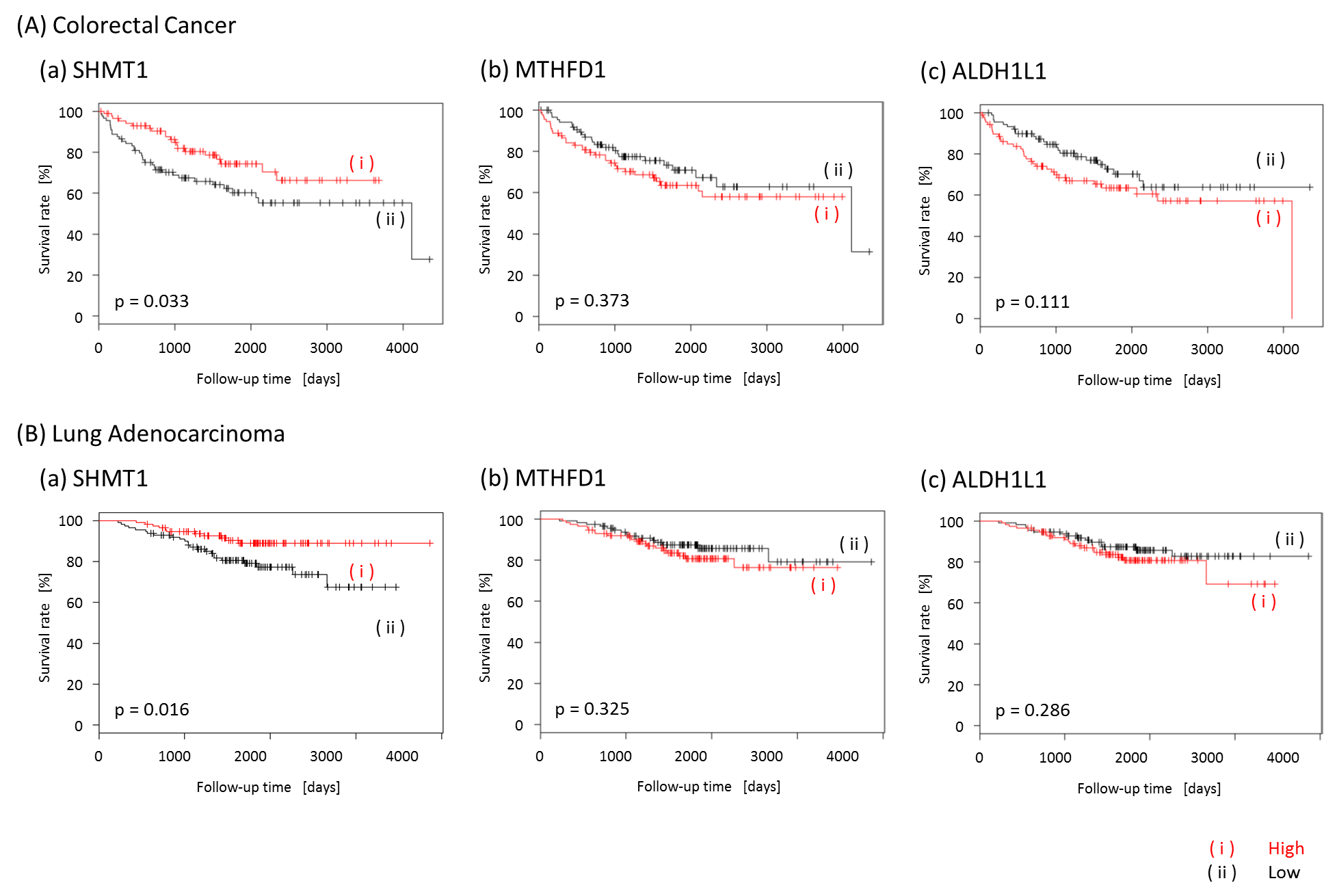


**Supplementary Figure 1.** Kaplan-Meier curves of overall survival for (A) Colorectal Cancer and (B) Lung Adenocarcinoma according to the expression of genes involved with cytoplasm specific folate metabolism (a) SHMT1, (b) MTHFD1, and (c) ALDH1L1.

**
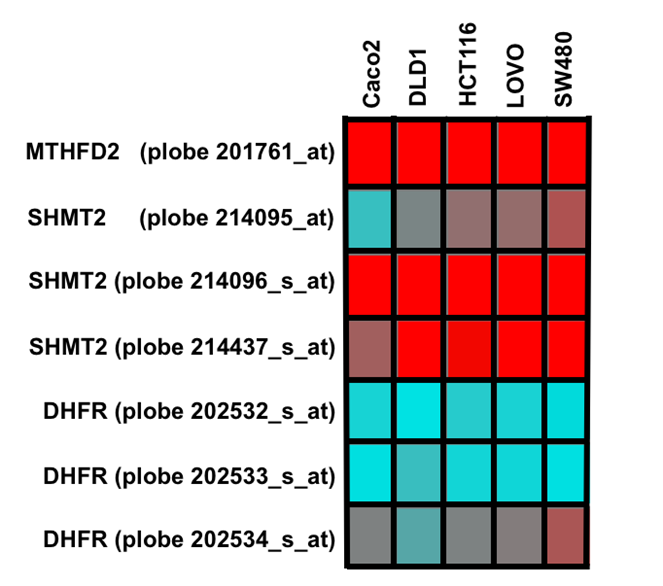
**

**Supplementary Figure 2.** Gene expression analysis for SHMT2, MTHFD2, and DHFR using RefExA database. The red areas indicate a high expression, and the blue areas indicate a low expression.

**
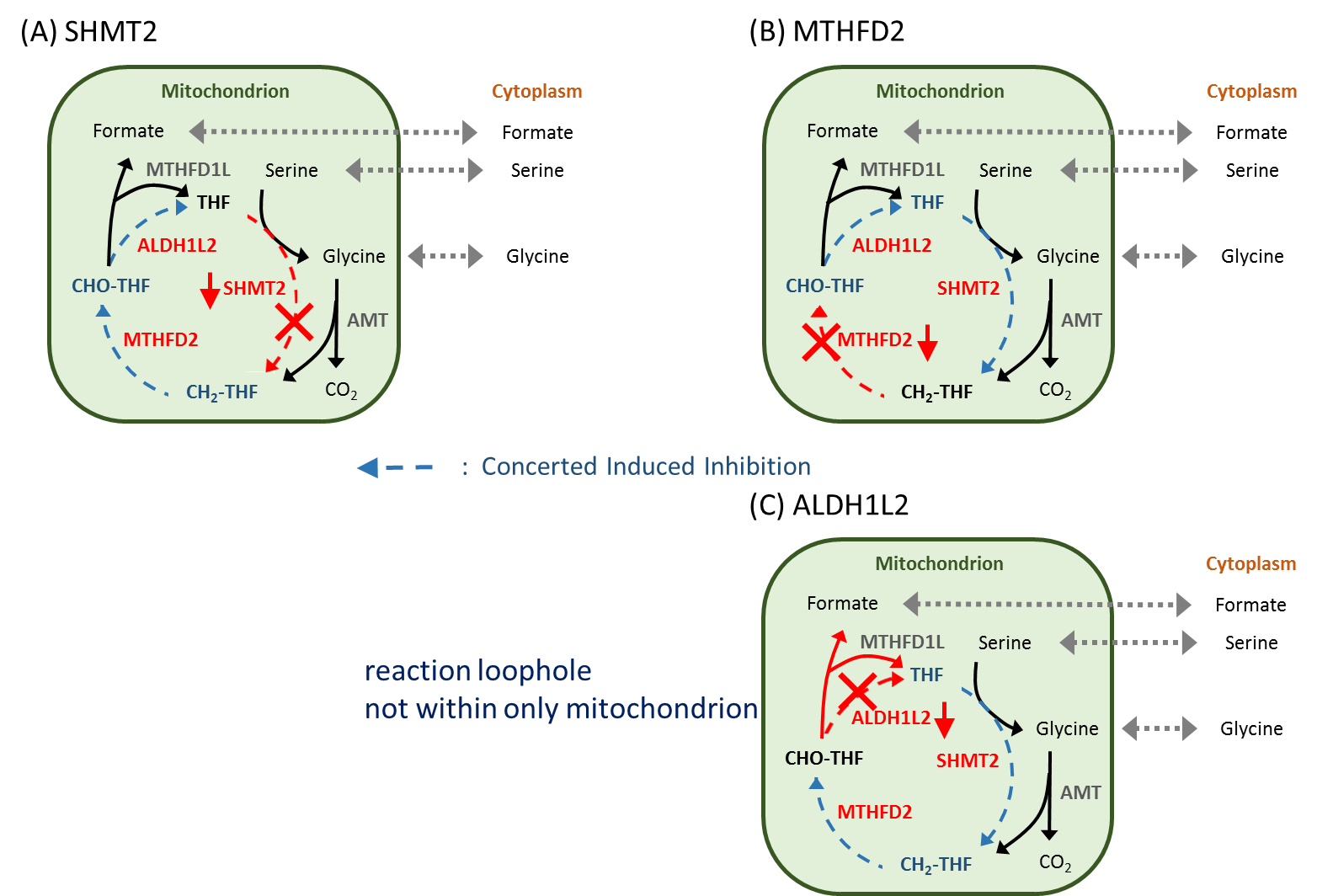
**

**Supplementary Figure 3.** A schematic diagram of the reaction changes via concerted induced inhibition.

**Supplementary Table 1.** Statistical tests for Correlation between both high group of “SHMT2 and MTHFD2”, and the other group.

1. Colorectal Cancer

|  | Distribution (High-High) / (Low-Low) | P-Value | Test |
| --- | --- | --- | --- |
| Age | 26-92 / 26-88 | 0.12 | t-test |
| Sex | M: 63 F: 42 / M: 33 F: 39 | 0.09 | χ^2^-test |

|  | Coefficient | p-value |
| --- | --- | --- |
| Cox proportional hazards | 1.8 | 0.032 |

|  |  | p-value |
| --- | --- | --- |
| Likelihood ratio test : | 4.51 | 0.034 |

1. Lung Adenocarcinoma

|  | Distribution (High-High) / (Low-Low) | P-Value | Test |
| --- | --- | --- | --- |
| Age | 30-76 / 38-71 | 0.50 | t-test |
| Sex | M: 58 F: 82 / M: 47 F: 39 | 0.07 | χ^2^-test |

|  | Coefficient | p-value |
| --- | --- | --- |
| Cox proportional hazards | 1.04 | 0.003 |

|  |  | p-value |
| --- | --- | --- |
| Likelihood ratio test : | 9.21 | 0.002 |

**Supplementary Table 2.** Statistical tests for Correlation between all high group of “SHMT2, MTHFD2 and ALDH1L2” and the other group.

1. Colorectal Cancer

|  | Distribution (All High) / (Other) | P-Value | Test |
| --- | --- | --- | --- |
| Age | 26-92 / 26-87 | 0.31 | t-test |
| Sex | M: 76 F: 51 / M: 20 F: 30 | 0.03 | χ^2^-test |

|  | Coefficient | p-value |
| --- | --- | --- |
| Cox proportional hazards | 0.626 | 0.027 |

|  |  | p-value |
| --- | --- | --- |
| Likelihood ratio test : | 4.57 | 0.033 |

1. Lung Adenocarcinoma

|  | Distribution (All High) / (Other) | P-Value | Test |
| --- | --- | --- | --- |
| Age | 30-76 / 45-71 | 0.69 | t-test |
| Sex | M: 72 F: 98 / M: 33 F: 23 | 0.05 | χ^2^-test |

|  | Coefficient | p-value |
| --- | --- | --- |
| Cox proportional hazards | 0.78 | 0.025 |

|  |  | p-value |
| --- | --- | --- |
| Likelihood ratio test : | 4.68 | 0.031 |
